# Ellis-van Creveld Syndrome: Mutations Uncovered in Lebanese Families

**DOI:** 10.1155/2015/528481

**Published:** 2015-04-30

**Authors:** Maria Valencia, Lara Tabet, Nadine Yazbeck, Alia Araj, Victor L. Ruiz-Perez, Khalil Charaffedine, Farah Fares, Rebecca Badra, Chantal Farra

**Affiliations:** ^1^Instituto de Investigaciones Biomédicas, Consejo Superior de Científicas, Universidad Autónoma de Madrid, Madrid, Spain; ^2^Department of Pathology and Laboratory Medicine, American University of Beirut Medical Center, P.O. Box 11-0236 Riad El Solh, Beirut 1107 2020, Lebanon; ^3^Department of Pediatrics and Adolescent Medicine, American University of Beirut Medical Center, P.O. Box 11-0236 Riad El Solh, Beirut 1107 2020, Lebanon; ^4^Centro de Investigación Biomédica en Red de Enfermedades Raras (CIBERER), Instituto de Salud Carlos III (ISCIII), Madrid, Spain

## Abstract

*Background*. Ellis-van Creveld (EvC) syndrome is a rare, autosomal recessive disorder characterized by short stature, short limbs, growth retardation, polydactyly, and ectodermal defects with cardiac anomalies occurring in around 60% of cases. EVC syndrome has been linked to mutations in *EVC* and *EVC2* genes. *Case Presentation*. We report EvC syndrome in two unrelated Lebanese families both having homozygous mutations in the *EVC2* gene, c.2653C>T (p.(Arg885^*^)) and c.2012_2015del (p.(Leu671^*^)) in exons 15 and 13, respectively, with the latter being reported for the first time. *Conclusion*. Although EvC has been largely described in the medical literature, clinical features of this syndrome vary. While more research is required to explore other genes involved in EvC, early diagnosis and therapeutic care are important to achieve a better quality of life.

## 1. Introduction

Ellis-van Creveld (EvC) syndrome, also known as chondroectodermal and mesoectodermal dysplasia, was first described in 1940 by Elis and Creveld, as an autosomal recessive disorder characterized by short stature and ribs, polydactyly, and ectodermal defects [1–4]. More than half of EvC patients manifest congenital heart defects [5].

While relatively rare, with a prevalence of 0.7/100,000 live birth and only around 300 cases reported worldwide [1, 3, 6], it is mainly reported in highly consanguineous populations such as the Amish population [7]. The syndrome can be diagnosed either by ultrasonography starting from 18th week of gestation or through clinical examination right after birth [3]. Diagnosis after birth is based on clinical observation of features and symptoms described above. It is also supported by an X-ray of the skeleton, chest radiography, ECG, and echocardiography [1].

EvC is related to a group of diseases with alteration of cilia (ciliopathies). Such abnormalities are caused by mutations in the EvC genes (*EVC* and* EVC2*) found on chromosome 4p16 [5, 8] in around two-thirds of the cases.

In this paper, we report EvC syndrome in two unrelated Lebanese families both having homozygous mutations in the* EVC2* gene, NM_147127.4: c.2653C>T p.(Arg885^*^) and NM_147127.4: c.2012_2015del (p.(Leu671^*^)) TAAT (p.(Leu671^*^)) in exons 15 and 13, respectively. While the first one has been recently reported in a Chinese patient, the latter is a newly described mutation.

Informed consents were obtained from the patients' guardians. Genetic analysis was approved by the Institutional Review Board at the Instituto de Investigaciones Biomédicas. Patients were clinically assessed by an experienced clinical geneticist.

**Figure 1:**
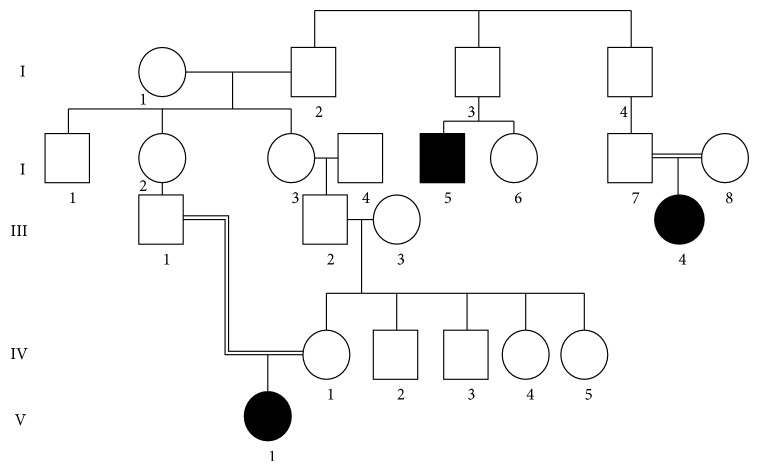
Pedigree of Family 1.

**Figure 2:**
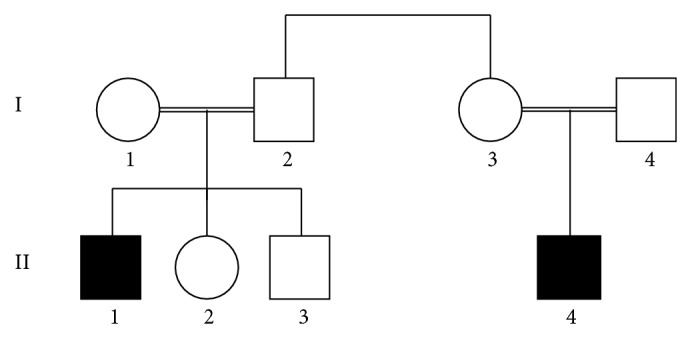
Pedigree of Family 2.

The pedigrees of families affected are shown in Figures 1 and 2.

## 2. Case Presentations

### 2.1. Family 1

A two-year-old girl born to healthy, young consanguineous parents was referred to our genetics clinic at the American University of Beirut Medical Center (AUBMC) for short stature. The patient had a trial septal defect for which she underwent surgical repair. There was no family history of similar problems and the pregnancy and delivery were reportedly uneventful.

**Figure 3:**
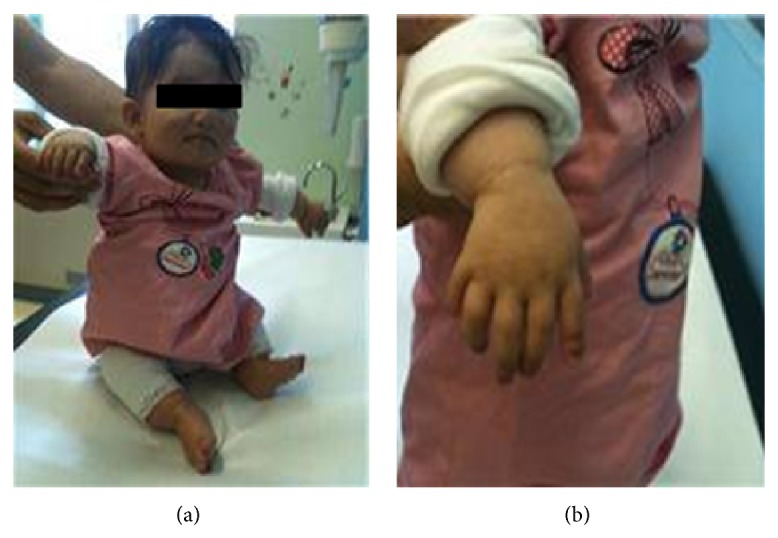
Clinical photos of patient from Family 1 depicting short upper and lower limbs and polydactyly.

Upon physical examination, both height and weight were on the 10th percentiles according to CDC Growth Charts (Ht = 79 cm; <10th percentile; wt = 10 kg; <10th percentile). Upper and lower segment ratio was 34/17 (Figures 3(a) and 3(b)).

Radiology results showed shortening of the paired long bones and slight elongation of the thorax with relatively short ribs, early ossification of femoral heads, and irregular acetabular roofs. The patient had bone deformities and polydactyly with short fingers.

Laboratory work-up yielded a normal 46XX karyotype but a high thyroid hormone level (TSH = 18.27 *μ*IU/mL) with free T4 within normal range. Gene sequencing showed a NM_147127.4: c.2653C>T (p.(Arg885^*^)) stop codon mutation in exon 15 of* EVC2*.

### 2.2. Family 2

A one-year-old boy born to first degree cousins was referred to our genetics clinic for assessment because of short stature and polydactyly. He was the second child of first cousin Lebanese parents.

The patient was delivered at term via C-section to a 35-year-old mother (G5P4). His birth weight was 2260 grams (<5%), with a length of 42 cm (<5%) and a head circumference of 33.3 cm (<25). An echo done at the 7th month of gestation showed a bell-shaped chest. No otherwise prenatal complications were reported. An echocardiogram done at birth showed Patent Foramen Ovale (PFO).

Upon physical examination the patient's stature was below the 5th percentile (height: 69 cm <5th%; wt: 20 cm <25th%). Radiology results showed shortening of the limbs and ribs.

**Figure 4:**
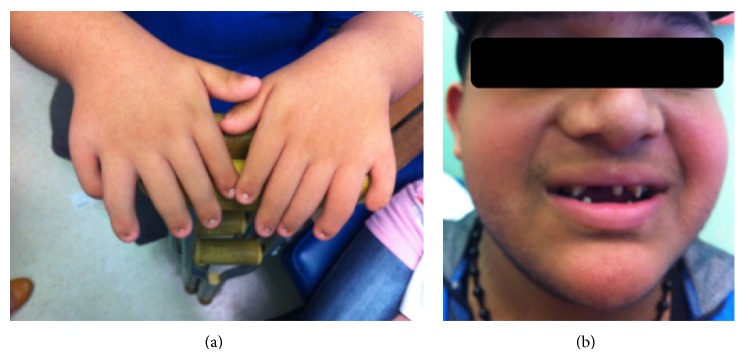
Clinical photos of patient from Family 2 showing conical shaped teeth and polydactyly.

He had a brother who passed away at day 5 of age and who was born with cleft lip and palate and bilateral polydactyly. One of his paternal cousins, a 17-year-old male with normal karyotype (46, XY), was reported to have polydactyly, short stature, and dysplastic nails and teeth. He was labelled as having dwarfism without proper follow-up or accurate assessment. Upon examination, he had widely spaced conical shaped teeth and a height of 140 cm (Figure 1; Figures 4(a) and 4(b)).

DNA analyses for* EVC* and* EVC2* genes on both cousins revealed a NM_147127.4: c.2012_2015del TAAT in exon 13 of* EVC2*.

## 3. Discussion

EvC is a rare disorder involving several embryonic tissues and resulting in polymorphic symptoms. Although largely described in the medical literature, the clinical features of this syndrome differ between patients [3, 7] with chondrodystrophia being the most commonly reported and the main reason behind short stature [1, 3, 5, 8].

Both families are consanguineous and presented with the classical findings of EvC syndrome. So far, around 93 mutations in either* EVC* or* EVC2* genes have been reported in the literature with the majority causing premature termination codons [5, 9, 10]. In both families, mutations were detected in* EVC2* gene. In the first family, a homozygous nonsense mutation c.2653C>T (p.(Arg885^*^)) was found. This mutation was previously reported in a 6-year-old Chinese female [10] who was a compound heterozygous for another mutation (IVS5-2A>G). Both our patient and the Chinese patient had similar clinical presentation [10]. In our second family, we detected a homozygous deletion of 4 nucleotides NM_147127.4: c.2012_2015del TAAT in exon 13 of* EVC2*. This deletion generates a frameshift that runs into a premature stop codon immediately. To our knowledge, this mutation is reported for the first time.

Amongst Arabs and Middle Eastern populations, mutation panels for recessive disorders in general differ from one country to another and in between religious denominations [11, 12] mainly because of the wide migratory movements that occurred over the centuries resulting in a great variability of ethnicity and origins that constitute these populations.

Lebanon is a small country with several ethnic and demographic groups originating in part, from European crusader Christians and Arabian Muslims, resulting in a heterogeneous background of our families [13]. Consanguineous marriages in Lebanon are relatively common (28.6%) leading to a high prevalence of autosomal recessive disorders [12, 14]. Despite this fact the need for genetic services in Lebanon is still not widely established. Indeed, lack of compliance for genetic referrals and testing is still witnessed mainly due to economic burdens since these tests do not benefit from third party coverage and also because of social taboos that are still associated with inherited diseases. For this reason, a substantial number of patients with genetic disorders may be misdiagnosed or not followed up properly. Reporting rare cases from our population will raise further awareness on the occurrence of these disorders and will increase the chance for a proper genetic assessment. This could also eventually contribute to unravel other genes that might be involved in EvC and to improve the quality of care for these patients.
